# Validation of an automated assay for measurement of bovine plasma ceruloplasmin

**DOI:** 10.1186/s13028-019-0470-4

**Published:** 2019-07-22

**Authors:** Hussein Awad Hussein, Jacqueline Bäumer, Rudolf Staufenbiel

**Affiliations:** 1 Veterinary Internal Medicine, Department of Animal Medicine, Faculty of Veterinary Medicine, Assiut University, Assiut, 71526 Egypt; 2 Klinik für Klauentiere, Freie Universität Berlin, 14163 Berlin, Germany

**Keywords:** Analytical, Automatic, Ceruloplasmin, Cows, Measurement, Validation

## Abstract

Ceruloplasmin (Cp) plays an important role in copper transport and iron metabolism, as well as Cp is also an indicator for the health status of dairy cows. The present study reports the validation of an automated assay to assess the plasma Cp in dairy cows. Plasma Cp levels were determined in 40 Holstein cows and intra- and inter-assay precision, accuracy, detection limit, and clinical validation of the assay were determined. Intra- and inter-assay coefficients of variation were < 2% and < 7%, respectively. The results were linear when serial sample dilutions were tested (*r* = 0.999, P < 0.001). The detection limit was lower than what could be measured in plasma from healthy cows. Increased plasma Cp levels were found in cows with inflammatory diseases. The method validated in this study is precise, simple, and fast and can be easily adapted to biochemical automated analysers. Furthermore, the promising results obtained with this protein will contribute to a wider use of Cp determination in bovine practice.

**Electronic supplementary material:**

The online version of this article (10.1186/s13028-019-0470-4) contains supplementary material, which is available to authorized users.

## Findings

Ceruloplasmin (Cp) is a protein ferroxidase that contains more than 95% of the copper present in plasma [1]. Cp plays many important functional roles such as copper transport and antioxidant properties [2].

Cp levels undergo physiological variations during different lactation stages [3] and it is also a mild to moderate acute phase protein that increases in levels in association with inflammation [4] and infection [5]. In dairy cows, Cp levels are indicative the animal’s health status [6] and it has the potential for being a marker of copper status [7, 8]. However, despite the importance and clinical significance that this protein could have in bovine practice only manual methods for Cp measurements have been validated in cows [9], while validation of automated assays for Cp measurement in bovine plasma has not been published. Therefore, the present study was performed for analytical validation of an automated assay for measuring Cp in plasma of dairy cows by investigating assay characteristics, including precision, accuracy, and detection limit. In addition, behaviour of the assay was evaluated in diseased cows.

Forty Holstein dairy cows were included in this study. All cows originated from a herd of the State Leipzig, Germany, were housed in a free stall, and fed a diet of grass and maize silage and concentrate as a totally mixed ration. The cows were randomly chosen. Based on a clinical examination made before sample collection, all cows were found clinically healthy, and their routine hematologic and biochemical test results were within normal reference range. Plasma levels of Cp was also measured in five diseased cows for validation of the assay in diseased animals. Diseased cows suffered from bronchopneumonia (n = 2), claw disorders and lameness (n = 2) and diarrhea with left displaced abomasum (n = 1). Values of Cp of diseased animals were compared with those obtained from the clinically healthy cows.

From each animal, one blood sample was obtained by puncture of the coccygeal vein and placed in a vacutainer tube containing lithium heparin as anticoagulant. Plasmas were harvested by centrifugation of blood samples at 2000*g* for 15 min.

The method employed has previously been published [8, 10] and is based on Cp’s ability to catalyse oxidation of *p*-phenylenediamine (PPD) to yield a purple colored product whose rate of formation is proportional to the concentration of Cp in the sample [10].

For the automated assay, 20 µL of plasma were mixed with 180 µL of sodium acetate buffer and 90 µL of PPD solution (Additional file 1). The oxidase activity of Cp was measured as the increase of absorbance per minute at 550 nm through the interval of 5 min (1st reading) and 20 min, 48 s. (2nd reading) after adding the reagents. The oxidase activity of Cp, which was indicated by the absorbance, was measured automatically using the Roche Cobas Mira Plus CC Chemical Analyzer (Roche Diagnostics, Bern, Switzerland) and expressed in g/L according to the following equation: Cp (g/L) = (A20.8 − A5) × 0.752.

For the manual assay, 100 μL of plasma was diluted with 2 mL 0.1 mol/L sodium acetate buffer and added to 1 mL freshly prepared buffered PPD solution, and then incubated at 37 °C. The absorbance reflecting the intensity of the purple colored product was measured by a spectrometer (Dr. Lange LP 700, Frankfurt, West Germany) at 550 nm, after 5 min (A5) and 30 min (A30) using 50 μL of sodium azide 1.5 mol/L for stopping the reaction. The manual assay of Cp was conducted as previously reported [8]. The oxidase activity of Cp was determined according to the following equation: Cp (g/L) = (A30 − A5) × 0.752.

In both assays, 0.752 is the calibration factor [10] and the biochemical reactions were carried out at 37 °C and protected from exposure to light. Table 1 summaries the comparison between the automatic and manual methods for Cp measurement.

**Table 1:** Comparison between automatic and manual assays for estimation of ceruloplasmin

Parameters	Automatic assay	Manual assay
Number and path of cuvette	One cuvette with ½ cm optical path	Two cuvettes with 1 cm optical path
Amount of the sample	20 µL	100 µL
Amount of sodium acetate buffer	180 µL	2000 µL
Amount of *p*-phenylenediamine buffer	90 µL	1000 µL
Amount of sodium azide 1.5 mol/L	NA	50 µL
Reaction time (consumed time till obtaining the results)	20.8 min	30 min

*NA* not applicable

Intra-assay precision was determined as the coefficient of variation (CV) between 10 replicates from two bovine plasma samples in one assay run. The same plasma samples were used to determine the inter-assay CV from the mean and standard deviation (SD) of 10 replicate determinations on 10 different days.

Accuracy was investigated by evaluation of linearity under dilution. To study linearity under dilution, plasma samples were diluted at 1:2, 1:4, 1:8, 1:16, 1:32, and 1:64 using sodium acetate buffer solution (0.1 mol/L, pH 5.45).

The limit of detection of the assay was determined as the lowest concentration of Cp in a sample that can be detected and was taken as the mean ± 2 SDs of 10 replicates of a reagent blank sample.

Data are presented as mean ± SD and the analysis was carried out using SPSS software. Intra- and inter-assay CVs were calculated by use of routine descriptive statistical procedures. Linearity under dilution was investigated by linear regression. Comparison between the automated and manual methods was assessed by paired sample *t*-test, Pearson correlation, Bland–Altman analyses and Passing-Bablok regression plot. An independent samples *t* test was performed to assess differences between healthy and diseased dairy cows. Significance was set at P < 0.05.

**Fig. 1:**
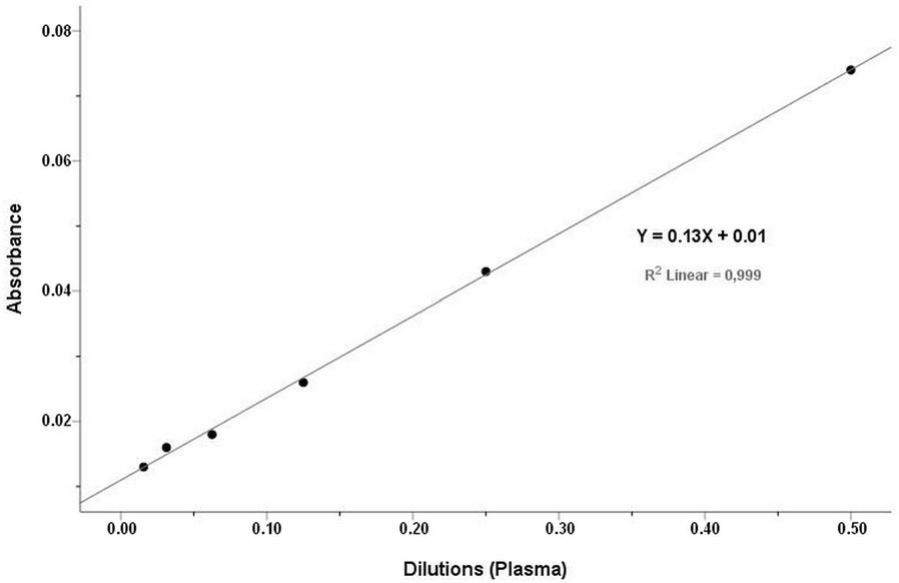
Linearity under dilution of plasma samples for determination of ceruloplasmin by automatic assay

**Fig. 2:**
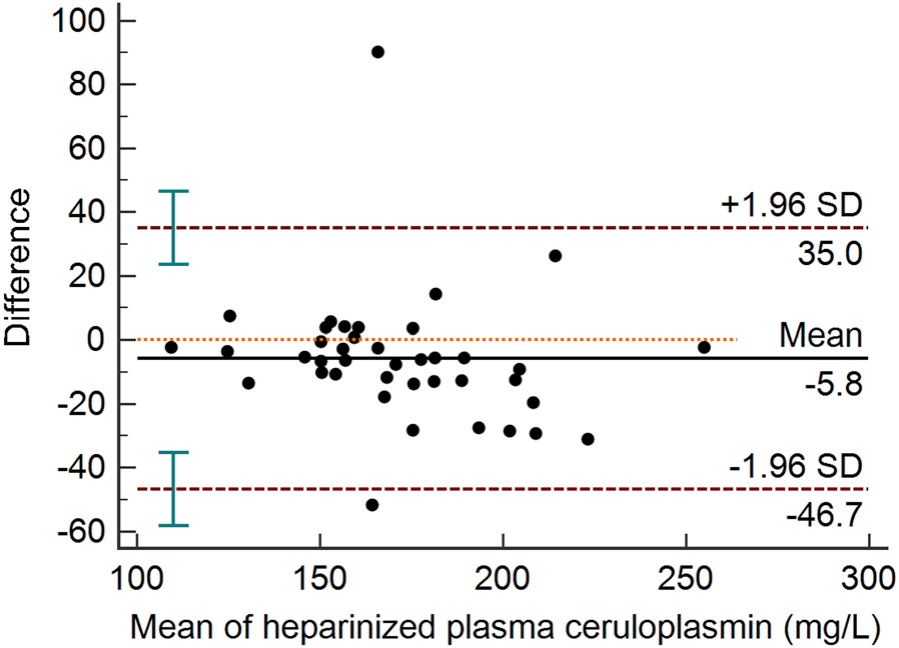
Bland–Altman difference plot for ceruloplasmin levels in bovine plasma samples (n = 40) using an automated and a manual assay. Y-axis: bovine plasma ceruloplasmin (BPCp) obtained with the automated assay minus BPCp value obtained with the manual method (Difference). X-axis: mean of the BPCp values obtained with the two methods. The mean of the differences or bias (line marked as mean) and the 95% limits of agreement (mean ± 1.96 SD) are included in the graph

**Fig. 3:**
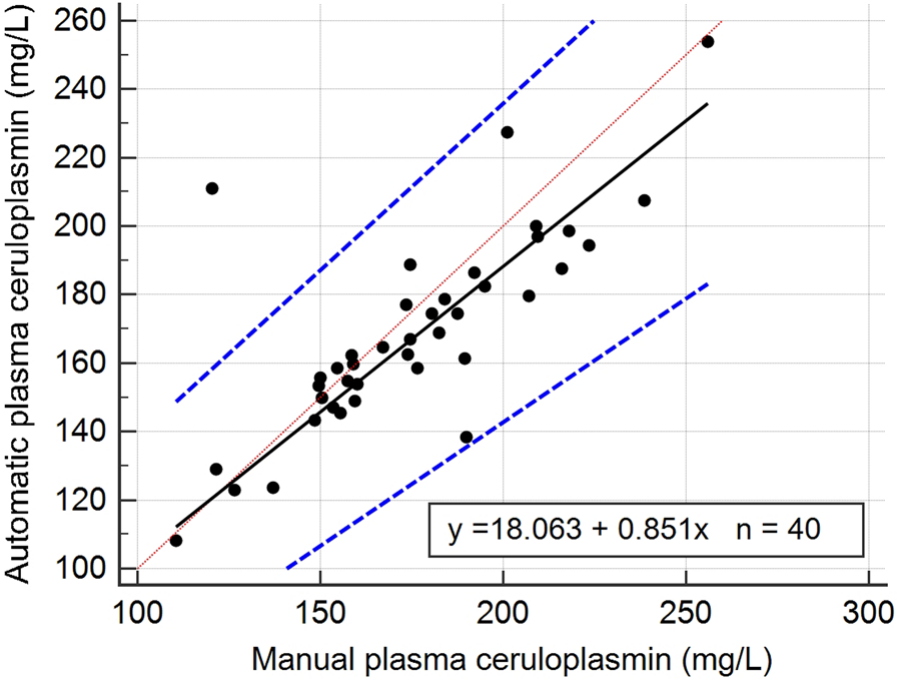
Passing Bablock regression plot for ceruloplasmin levels in bovine plasma samples (n = 40) using automatic and manual assays. Black line represents the regression line, the 2 dashed blue lines represent the 95% confidence interval, and the dotted red line is the identity line (Y = X)

**Fig. 4:**
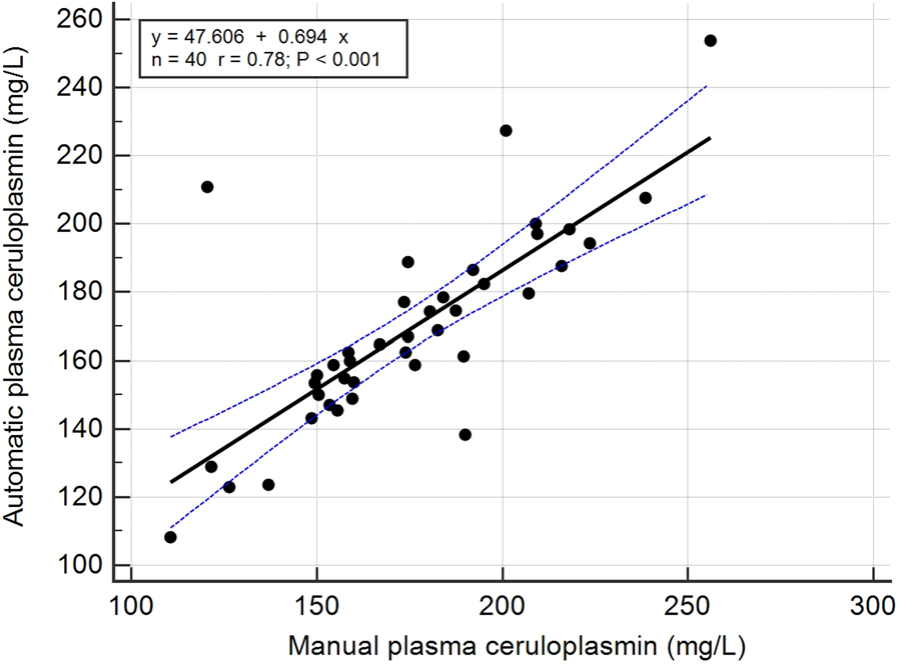
Scatter plot for the regression analysis between manual and automatic assays. Values obtained with the 2 methods were plotted and adjusted to a regression line (black line). The dashed blue lines represent the 95% confidence interval curves

**Fig. 5:**
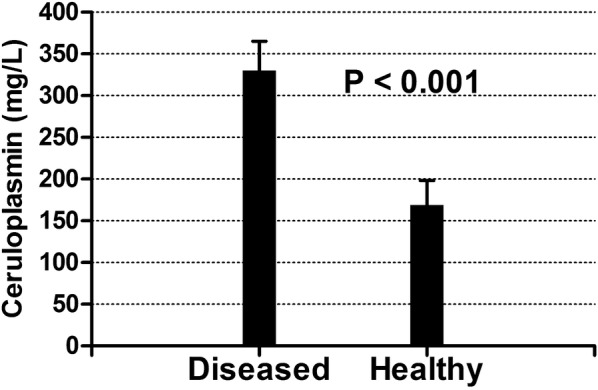
Levels of ceruloplasmin in diseased and healthy cows. The plots show mean ± SD

The mean, SD, and the intra- and inter-assay CVs obtained with the automated assay are shown in Table 2. Intra-assay CVs were lower than 2% and the inter-assay CVs were lower than 7%. Serial dilution of a plasma sample resulted in a linear regression equation with an *r*^*2*^ value of 0.999 (Fig. 1). Limit of detection of the assay was 7.27 mg/L and the Bland–Altman plot indicated a proportional discrepancy between the manual and automated assays (bias = − 5.8, SD = 20.82; Fig. 2). Passing-Bablok regression analysis (Fig. 3) gave an intercept of 18.06 (95% confidence interval (CI) − 1.78 to 41.05) and a slope of 0.85 (95% CI 0.72 to 0.97), suggesting a proportional difference between the two methods. The custom test showed no significant deviation from linearity (P = 0.80). The relationship between the manual and automated assays was determined and illustrated in Fig. 4. A correlation coefficient (*r*) of 0.78 was obtained. Figure 5 shows the plasma values of Cp in healthy and diseased dairy cows. The level of Cp was significantly higher in diseased cows than healthy ones (330 ± 35 vs. 169 ± 29; P< 0.001).

**Table 2:** Intra- and inter-assay coefficients of variations (CVs) for 10 replicates of 2 plasma samples for precision analysis of ceruloplasmin using an automated assay

Samples	Series	Intra-assay		Inter-assay	
		Mean ± SD	CV (%)	Mean ± SD	CV (%)
Plasma	Series 1	97 ± 1.02	1.06	152 ± 4.38	2.87
	Series 2	237 ± 4.74	2.00	216 ± 14.27	6.59

Although automated methods were validated for measurement of Cp in canine [12] and porcine [13] blood, these studies used either different buffer, pH and/or different substrates for the assays. In the present research, validation of the automated assay showed a good precision with intra- and inter-assay CVs lower than 7%. The intra-assay CV was lower than that obtained by Cerón and Martínez-Subiela [12] for canine serum; a difference that may be attributed to sample, technique, or animal species variations. In a previous study [6], Cp levels in serum samples were approximately 30% lower relative to heparinized plasma samples. In the current study, inter-assay CV was higher than those obtained by Hussein and Staufenbiel [9] using a manual method for Cp measurement. Such variation in the CV could contribute to the automation of the assay.

The automated method showed a good linearity with serial dilutions of bovine plasma samples. The linearity under dilution revealed high regression coefficients, indicating that the assay measured Cp in a linear manner with a detection limit of 7.27 mg/L. The assay was linear and showed a proportional bias with the manual assay. This proportional discrepancy between both assays could be explained as a technique variation. In a previous study [13], the authors found a proportional bias between two automated assays for Cp measurement in pigs. Here in comparison with the manual assay, small amount of plasma was used with a minimum reaction time in the automated assay. Furthermore, the automated assay has no hazard of toxicity by sodium azide, as well as no human factor errors may exist. However, a strong correlation was found between the automated and manual assays.

Despite the number of diseased cows was somewhat limited; the values of Cp were higher than in healthy cows. Such increase may be due to the inflammatory and oxidative stress processes in diseased animals, supporting the results of previous studies that revealed increased Cp in diseased cows [5, 11]. Furthermore, Cp is a mild to moderate acute phase protein that increases in association with inflammation [14]. However, a further research study including much greater number of diseased cows may be required.

In conclusions, the method for Cp measurement shows adequate analytical precision and accuracy. It is cheap and easy to adapt to other automated biochemical analyzers, considerably decreasing the processing time required with the manual method. It is expected that the description of this method and the promising results obtained with this protein in clinical trials will contribute to a wider use of Cp determination in bovine practice.

## Additional file

**Additional file 1.** Preparation of the reagents’ solutions for the biochemical assays.

## Data Availability

The datasets during and/or analyzed during the current study are available from the corresponding author on reasonable request.

## Abbreviations

Cp: ceruloplasmin
CV: confidence intervals
PPD: *p*-phenylenediamine
SPSS: statistical package for the social sciences
