# Supplementary material for: Validation of an automated assay for measurement of bovine plasma ceruloplasmin
Source: Acta Vet Scand. 2019 Jul 22;61:34. doi: 10.1186/s13028-019-0470-4 (PMC6647283; doi:10.1186/s13028-019-0470-4)
Supplement: Supplementary file 1 — Additional file 1. Preparation of the reagents’ solutions for the biochemical assays. [file 13028_2019_470_MOESM1_ESM.doc]

**Additional file 1.** Preparation of the reagents' solutions for the biochemical assays.

- Sodium acetate solution (0.2 mol/L): it was prepared by dissolving of 16.4 g of sodium acetate (VWR Chemicals, Germany) in a liter of distilled water.
- Acetic acid solution (0.2 mol/L): it was prepared by adding 12 mL of acetic acid 96% in a liter of distilled water.
- Sodium acetate buffer solution (0.1 mol/L, pH 5.45) at 37 ºC: into a one-liter volumetric flask are transferred 430 ml of sodium acetate solution, 0.2 mol/L; 70 ml of acetic acid solution, 0.2 mol/L; and 400 ml of distilled water. The contents are warmed to 37 ºC in a water bath, the pH was adjusted to 5.45 by addition of sodium acetate or acetic acid, and then the contents of the flask were diluted to the mark with distilled water.
- Sodium azide solution (1.5 mol/L): it was prepared by dissolving 9.75 g of sodium azide (Merck, Germany) in 100 mL of distilled water.
- *p*-phenylenediamine (PPD) buffer solution (27.6 mmol/L): in a 100-ml volumetric flask, dissolving of 0.5 g of *p*-phenylenediamine dihydrochloride (Sigma-Aldrich, Germany) in 75 ml of sodium acetate buffer solution (0.1 mol/L, pH 5.45, 37 ºC). The content of the flask was well mixed and adjusted to pH 5.45 at 37 ºC by dropwise addition of sodium hydroxide 2N, and diluted to the mark with sodium acetate buffer solution.
